# The development of drugs for treatment of sleeping sickness: a historical review

**DOI:** 10.1186/1756-3305-3-15

**Published:** 2010-03-10

**Authors:** Dietmar Steverding

**Affiliations:** 1 BioMedical Research Centre, School of Medicine, Health Policy and Practice, University of East Anglia, Norwich NR4 7TJ, UK

## Abstract

Only four drugs are available for the chemotherapy of human African trypanosomiasis or sleeping sickness; Suramin, pentamidine, melarsoprol and eflornithine. The history of the development of these drugs is well known and documented. suramin, pentamidine and melarsoprol were developed in the first half of the last century by the then recently established methods of medicinal chemistry. Eflornithine, originally developed in the 1970s as an anti-cancer drug, became a treatment of sleeping sickness largely by accident. This review summarises the developmental processes which led to these chemotherapies from the discovery of the first bioactive lead compounds to the identification of the final drugs.

## Background

Human African trypanosomiasis or sleeping sickness is a disease caused by two subspecies of *Trypanosoma brucei*, *T. b. rhodesiense *and *T. b. gambiense*. The parasites live and multiply extracellularly in blood and tissue fluids of their human host and are transmitted by the bite of infected tsetse flies (*Glossina *spp.). The occurrence of sleeping sickness is restricted to the distribution of tsetse flies which are exclusively found in sub-Saharan Africa between 14°N and 20°S [1]. More than 250 discrete active sleeping sickness foci in 36 African countries are recognised most of which are in rural areas [2].

*Trypanosoma b. rhodesiense *is found in East and southern Africa whereas *T. b. gambiense *occurs in West and Central Africa. The course of sleeping sickness is different depending on the subspecies. Infections with *T. b. rhodesiense *lead to an acute form of the disease while infections with *T. b. gambiense *give rise to a chronic infection. The symptoms of the first stage of the disease, defined by the restriction of trypanosomes to the blood and lymph system, include fever, headache, joint pain and itching [3,4]. The clinical signs of the second stage of the disease, characterised by the invasion of trypanosomes into the central nervous system, are neurological and endocrinal disorders [3,4]. If left untreated, sleeping sickness patients infected with *T. b. rhodesiense *will die within months whereas those infected with *T. b. gambiense *usually survive for several years.

In the late 19th Century, Africa experienced several sleeping sickness epidemics the most devastating of which was an epidemic with 300,000 to 500,000 deaths between 1896 and 1906 which mainly affected the Congo Basin and the Busoga focus in Uganda and Kenya [5]. The disastrous effect of this epidemic persuaded the various colonial administrations to call for their medical scientists to develop a cure for sleeping sickness. At that time, the field of chemotherapy was developing and had begun to make use of the novel methods of medicinal chemistry, i.e. the identification, synthesis and development of new chemical entities suitable for therapeutic use. In fact, it was for the development of early anti-sleeping sickness drugs that medicinal chemistry was first used [6,7].

## Dyestuffs

**Figure 1:**
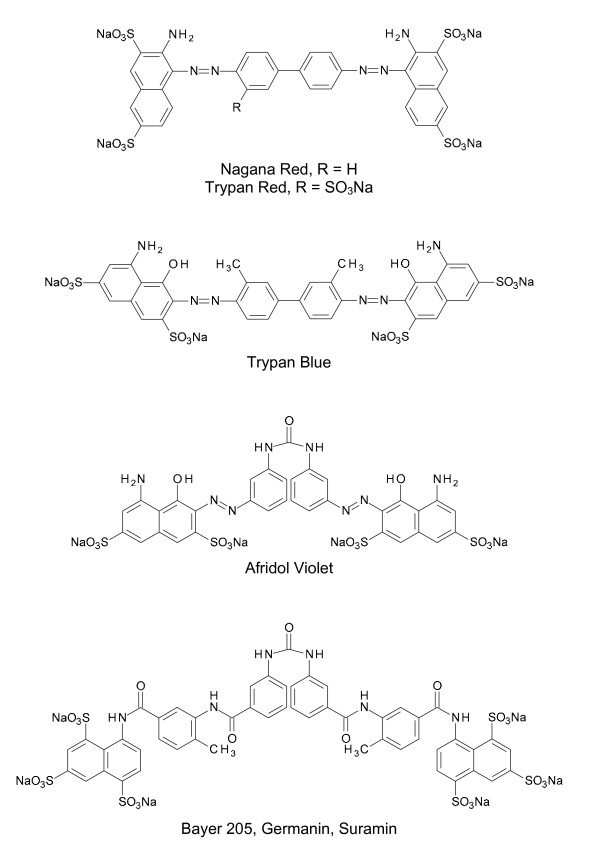
**Chemical structures of sulphated naphthylamine derivatives with trypanocidal activities**.

The synthetic dyestuff industry evolved in the middle of the 19th Century, primarily in Germany which became the world leader [8]. The most influential protagonist of the synthetic dyestuff industry was the German scientist Paul Ehrlich who was the first to exploit the properties of dyes for the development of chemotherapies. In 1901, Ehrlich became interested in the chemotherapy of trypanosomiasis and tested more than 100 synthetic dyes against *Trypanosoma equinum*, a species that causes a disease known as Mal de Caderas in equids, and *T. brucei brucei*, which is responsible for Nagana, a disease of cattle [9]. The only dye displaying trypanocidal activity was a benzopurpurine compound named Nagana Red (Fig. 1). When trypanosome-infected mice were treated with Nagana Red, parasites became undetectable in the animals for a short period and the treated mice survived 2 days longer than control mice (5/6 days versus 3/4 days). Ehrlich thought that the poor efficacy of Nagana Red was due to its low solubility which impaired the absorption of the drug into the bloodstream from the subcutaneous inoculation site. In 1903, Ludwig Benda working for Cassella Farbwerke near Frankfurt synthesised a derivative of Nagana Red, called Trypan Red (Fig. 1), with an extra sulphonic acid function and enhanced water solubility [10]. Trypan Red proved to be both curative and prophylactic for *T. equinum *infections in mice [11] but not for infections with other trypanosome species [12]. Ehrlich investigated another 50 derivatives of Trypan Red and the 7-amino derivative of the compound was tested by the German physician Robert Koch during an expedition in 1906 [9]. However, the 7-amino derivative was not more effective than Trypan Red itself.

From 1906, the German Bayer pharmaceutical company supplied the French bacteriologist Maurice Nicolle and the French zoologist Felix Mesnil from the Pasteur Institute with benzopurpurine dyes to be tested for trypanocidal activities [10]. One blue benzopurpurine derivative, Trypan Blue (Fig. 1), was found to be very effective in eliminating all trypanosomes from the blood of infected animals [13,14] but as the drug stained the skin of the animals bluish it was unacceptable for use in patients [9,10]. For this reason Wilhelm Roehl a former assistant of Ehrlich who had joined the Bayer research group at Elberfeld in 1905, sought a colourless compounds with trypanocidal activities. The chemists Oskar Dressel and Richard Kothe of Roehl's team synthesised derivatives of Afridol Violet (Fig. 1), a naphthalene urea compound that was less colour-intensive but also less trypanocidal in screens carried out by Nicolle and Mesnil. Several derivatives, however, displayed better activity against trypanosomes than the parent compound. In 1917, after the synthesis and screening of more than 1000 naphthalene ureas, the breakthrough came in the form of Bayer 205 (Fig. 1), later named Germanin, a colourless compound that cured trypanosomiasis in both experimental animals and in humans [15]. The Bayer Company understood the political importance of Bayer 205 for the commercial exploitation of African colonies and offered the formula of the drug to the British Government in exchange for the return of Germany's lost African territories [16]. When the British declined the offer, the Bayer Company refused to disclose the chemical structure of the drug. Eventually, in 1924, the French pharmacist Ernest Fourneau published the structure of Bayer 205 [17]. Four years later, the Bayer Company confirmed that Fourneau's structure was identical with that of Germanin. The drug was later renamed suramin and is still in use in the therapy of early-stage *T. b. rhodesiense *sleeping sickness.

## Arsenicals

**Figure 2:**
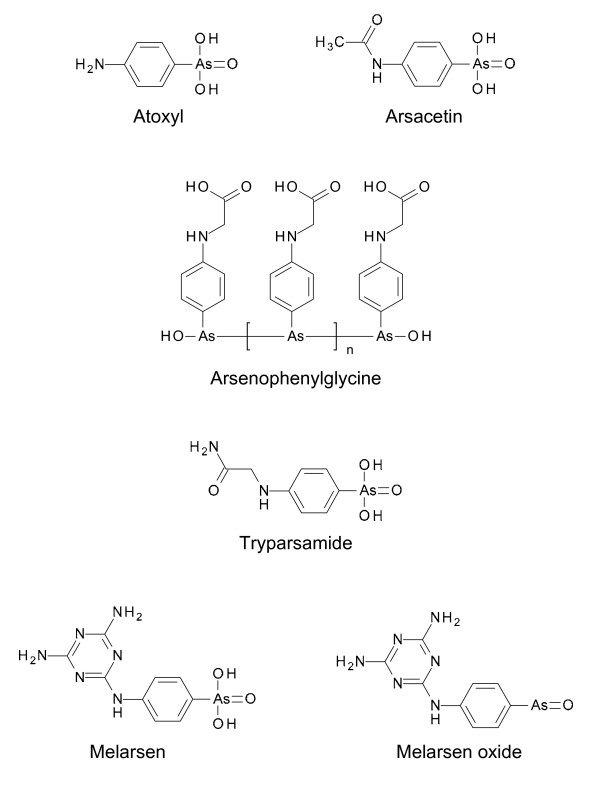
**Chemical structures of main arsenical compounds with trypanocidal activities**. With respect to arsenophenylglycine, Ehrlich thought that it consisted of two molecules joined by a As = As double bond. Years later it was found that arsenophenylglycine and other arsenobenzenes are polymers of several molecules whose arsenic atoms were linked by single bonds [9].

In 1858, the Scottish missionary and explorer David Livingston had suggested the use of Fowler's Solution, a 1% aqueous solution of potassium arsenite, for the treatment of sleeping sickness [18], although the causative agent of the disease was still unknown. In 1902, the French physician Charles Louis Alphonse Laveran (who had discovered the malaria parasite in 1880) together with Mesnil reported that sodium arsenite was effective in killing trypanosomes in infected laboratory animals. Although the parasites were quickly destroyed by sodium arsenite, they reappeared in the blood of the animals within a few days and caused their death [19]. Two years later, the Canadian doctor Harold Wolferstan Thomas published a paper describing the successful therapy of animals experimentally infected with trypanosomes with the arsenical drug Atoxyl (aminophenyl arsonic acid; Fig. 2) [20]. The drug had already been synthesised in 1859 by the French biologist Antoine Béchamp and was claimed to be 40-50 times less toxic than arsenic acid [9]; hence the name Atoxyl. The original structure of Atoxyl, arsenic acid anilide, assigned by Béchamp was incorrect and it was Ehrlich and Alfred Bertheim, Ehrlich's chief organic chemist, who determined the correct structure as an amino derivative of phenyl arsenic acid [6] which was instrumental in the generation of new analogues (see below). During a trial to evaluate Atoxyl on sleeping sickness patients in East Africa, Robert Koch discovered that the compound was by no means nontoxic: about 2% of treated patients went blind through atrophy of the optic nerve [9]. Based on this finding, Koch asked Ehrlich to synthesise derivatives of Atoxyl with reduced toxicity and improved efficacy. Bertheim synthesised a series of N-substituted Atoxyl derivatives. One such compound, acetylatoxyl or arsacetin (Fig. 2), was less toxic to mice, but when given in the high doses needed for therapy, the animals began to move uncontrollably in circles indicating that the vestibular nerve was damaged [6]. This observation suggested that arsacetin might also cause blindness. In addition, Ehrlich found that arsacetin was ineffective *in vitro *and therefore assumed that the compound was metabolised into an active form, probably by a chemical reduction. To test his hypothesis, he asked Bertheim to synthesise the two possible types of reduction products from Atoxyl derivatives: arsenoxides and arsenobenzenes. Whereas the arsenoxides were toxic to both trypanosomes and the host, arsenobenzenes were less trypanocidal but still more potent than arsenoxides and less cytotoxic [6,9]. This meant that arsenobenzenes could be administrated at sufficiently low doses to avoid the neurotoxicity problems. The most promising arsenobenzene turned out to be arsenophenylglycine (Fig. 2). When tested in humans in 1907 it proved to be safe and effective, except for a few patients who developed a severe and often fatal hypersensitivity reaction [6,9].

Ehrlich obtained another arsenobenzene, arsenophenol, by inserting a hydroxyl group in *para *position of the benzene ring of Atoxyl followed by reduction. This compound was highly effective against trypanosomes but prone to oxidation and difficult to purify. Based on his experience with chemotherapeutic dyes, Ehrlich knew that the addition of a substituent in *ortho *position to the hydroxyl group would enhance the chemotherapeutic activity. The introduction of an amino group led to the compound 606 or arsphenamine, which was synthesised by Bertheim in 1907 but which turned out to be ineffective against trypanosomes [6,9]. Interestingly, however, in 1909 Ehrlich and his colleague Sahachiro Hata, a Japanese bacteriologist, discovered that arsphenamine had excellent curative properties against syphilis. It became the first truly effective drug for treatment of the disease and was marketed by Farbwerke Hoechst under the proprietary name Salvarsan, 'the arsenic that saves' [6].

In 1919, the American chemist Walter Jacobs and the American immunologist Michael Heidelberger reported the synthesis of tryparsamide (Fig. 2), a derivative of Atoxyl [21]. As this compound was able to enter the central nervous system it was the first drug for treating the second stage of sleeping sickness alone or in combination with suramin. Although tryparsamide also caused damage to the optical nerve, it remained the drug of choice for chemotherapy of sleeping sickness until the early 1960s [21] and was also used in the treatment of animal trypanosomiasis [22].

**Figure 3:**
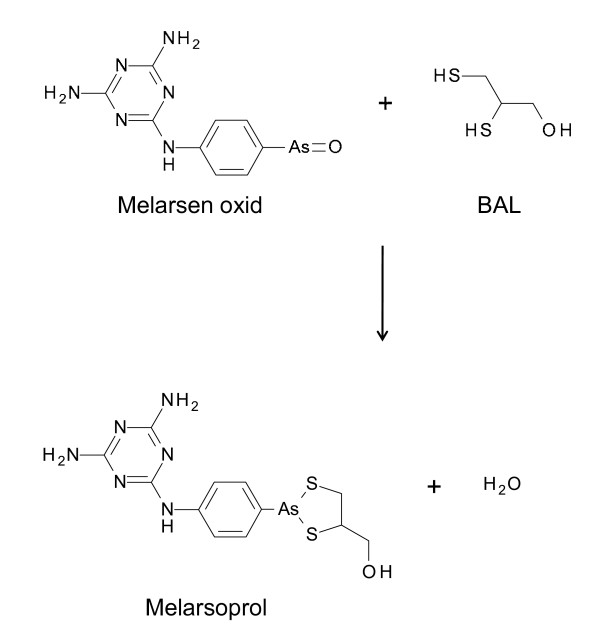
**Reaction of melarsen oxide with British anti-Lewisite (BAL) to produce melarsoprol**.

In 1938, the Swiss pathologist, microbiologist and chemist Ernst Friedheim synthesised melarsen (Fig. 2), a melamine derivative of Atoxyl [23]. Friedheim introduced the melamine moiety because he observed that all arsenicals with significant trypanocidal activity contain nitrogen in one form or another [24]. Although melarsen was less toxic than existing drugs, it was more expensive. Bearing in mind that trivalent arsenicals are much more potent than pentavalent arsenicals, Friedheim synthesised the trivalent analogue of melarsen, melarsen oxide (Fig. 2). This was very effective against trypanosomes but also very toxic [25]. Based on the observation that a diet of Swiss cheese improved the tolerability of melarsen oxide in some patients [24], Friedheim had the brilliant idea to combine the drug with dimercaprol, an arsenic antidote also known as British anti-Lewisite (BAL) developed during World War II to protect against poisoning by the arsenic gas, lewisite. The resulting product, melarsoprol (also known as Mel B) (Fig. 3), was 100 times less cytotoxic but only 2.5 times less trypanocidal than melarsen oxide [26]. Melarsoprol was introduced in 1949 for the treatment of sleeping sickness. The major advantage of melarsoprol over tryparsamide is that it does not show any toxic effect on the optic nerve. However, melarsoprol causes reactive encephalopathy in 5-10% of patients, with 1-5% mortality [3]. Melarsoprol is still the only effective drug for chemotherapy of second stage of *T. b. rhodesiense *sleeping sickness.

## Diamidines

**Figure 4:**
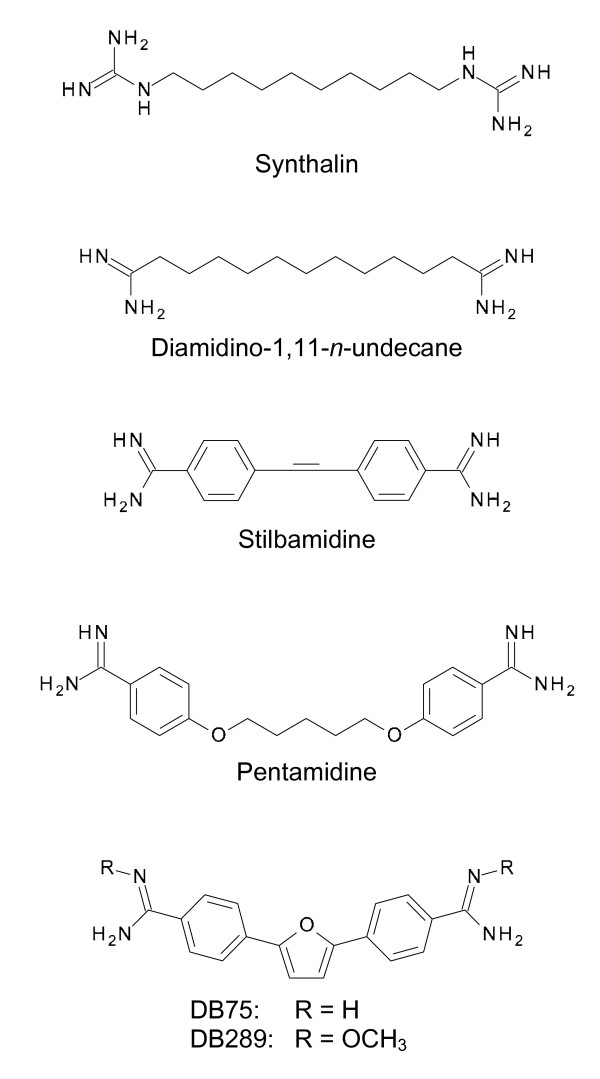
**Chemical structures of diamidines with trypanocidal activities**.

In 1929 it was shown for the first time that pathogenic trypanosomes use enormous amounts of sugar for their metabolism [27]; within 24 h they consume about twice their own mass of sugar. In 1935, this knowledge prompted the American bacteriologist Hildrus Poindexter to treat animals infected with *Trypanosoma equiperdum*, a trypanosome that causes Dourine or covering disease in horses, with insulin to reduce their blood glucose levels. He discovered that trypanosome-infected animals subjected to insulin treatment survived longer and with fewer parasites in the blood compared to controls [28]. In the same year, von Jancsó and von Jancsó and Schern and Artagaveytia-Allende independently found that the hypoglycaemic drug synthalin (Fig. 4) had trypanocidal action in mice and rats [29,30]. In 1937, Yorke and Lourie at the Liverpool School of Tropical Medicine discovered that the anti-trypanosomal effect of synthalin had nothing to do with lowering the blood glucose level in the infected animals but that synthalin itself was trypanocidal [31]. When Harold King at the National Institute of Medical Research in London learnt about the trypanocidal action of synthalin, he synthesised and tested related compounds and found that diamidino-1,11-*n*-undecane (Fig. 4) was particularly active against *T. b. rhodeseinse *in mice [32]. Meanwhile, the English chemist Arthur James Ewins of the pharmaceutical company May and Baker prepared a large number of aromatic diamidines in which the polar amidine groups were separated by two phenyl groups rather than by a polymethylene group. Many of these compounds displayed trypanocidal activity, especially stilbamidine and pentamidine (Fig. 4) [33,34]. Both compounds were also highly effective against human trypanosomiasis [35]. Whereas stilbamidine was later abandoned, because it caused serious neurological effects in some patients [36,37], pentamidine is still used for treatment of the first stage of *T. b. gambiense *sleeping sickness. Pentamidine is also used in the treatment of leishmaniasis and *Pneumocystis jirovecii *pneumonia, mostly in AIDS patients [38].

In 1977, Das and Boykin reported on the trypanocidal activity of a series of novel aromatic diamidines, with DB75 (2,5-bis(4-aminophenyl)-furan; Fig. 4) as the most trypanocidal compound active against *T. b. rhodesiense *in mice and rhesus monkeys [39,40]. As DB75 is poorly absorbed across the gastrointestinal tract due to its positively charged amidine group, a prodrug, DB289 (2,5-bis(4-amidinophenyl)-furan-bis-*O*-methylamidoxime; Fig. 4), was synthesised [41]. DB289 was the first oral drug for treatment of first-stage sleeping sickness to enter clinical trials [42]. However, an extended phase I study to complete the safety assessment for registration of DB289 for sleeping sickness revealed severe liver toxicity and delayed renal insufficiency [42]. As a consequence, the program to develop DB289 as an oral drug for treatment of sleeping sickness was discontinued [42].

## Nifurtimox

**Figure 5:**
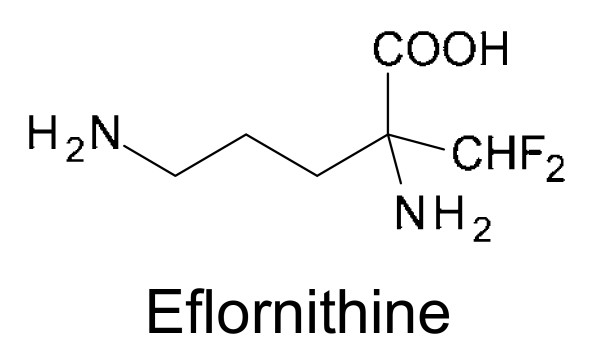
**Chemical structure of nifurtimox**.

Nifurtimox, a nitrofuran derivative (Fig. 5), was developed in the 1960s by the Bayer Company. Its trypanocidal activity was empirically discovered and since 1967 it has been used for the treatment of Chagas disease caused by *Trypanosoma cruzi *in Latin America. The mode of action of nifurtimox is not clearly known but may be related to the generation of reactive oxygen species which damage cellular components such as DNA, membrane lipids and proteins [43]. Nifurtimox was also tried on compassionate grounds for treatment of sleeping sickness patients who were infected with melarsoprol-resistant *T. b. gambiense*. Small clinical trials showed that high dosage of nifurtimox was necessary to achieve a cure [44,45]. More recently, nifurtimox has been evaluated together with eflornithine as combination therapy for treatment of late-stage *T. b. gambiense *sleeping sickness (see below).

## DFMO

**Figure 6:**
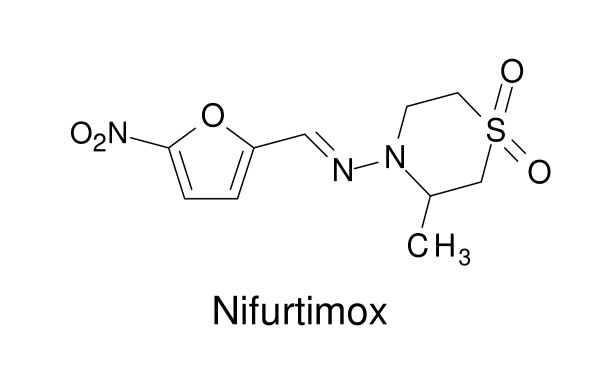
**Chemical structure of eflornithine**.

Eflornithine (α-difluoromethylornithine, DFMO, Fig. 6) was initially developed in the 1970s [46] as a potential anti-cancer drug [47]. Eflornithine was one of several fluorinated analogues of amino acids that were rationally designed based on a predicted enzyme reaction to generate mechanism-based inhibitors of amino acid decarboxylase. It is a specific and irreversible suicide inhibitor of ornithine decarboxylase [46,48], the first enzyme involved in polyamine biosynthesis. Inhibition of ornithine decarboxylase results in the depletion of the polyamines putrescine and spermidine, with the consequence of a slowdown in cell proliferation. It also causes a loss of the unique antioxidant metabolite, trypanothione, which may also play a role in its selective action and also accounts for the known synergism with other drugs [49]. The very rapid turnover rate of mammalian ornithine decarboxylase (t_1/2 _= 10-30 min [48,50]), however, made DFMO ineffective as an anti-cancer drug.

In 1980, the American biologist Cyrus Bacchi heard about DFMO and tested the drug in a murine trypanosomiasis model and showed that DFMO cured mice infected with a virulent strain of *T. b. brucei *without any apparent toxic side effects [51]. Based on this remarkable result, several clinical trials were carried out demonstrating that DFMO can cure second-stage *T. b. gambiense *sleeping sickness patients who were refractory to melarsoprol treatment [52-55]. The reason why DFMO is only active against *T. b. gambiense *is that the ornithine decarboxylase of this trypanosome species is very stable (t_1/2 _= 18-19 h) [56]. In contrast, the DFMO tolerance of *T. b. rhodesiense *is due to a faster turnover rate of the enzyme (t_1/2 _= 4.3 h) [56]. In 1990, DFMO was approved for the treatment of human trypanosomiasis caused by *T. b. gambiense *[57] and is currently the only treatment available for melarsoprol-refractory sleeping sickness.

In recent years, eflornithine has been tested in combination with nifurtimox in the treatment of second-stage *T. b. gambiense *sleeping sickness [58-60]. A recently completed multicentre, randomised, phase III trial revealed that the efficacy of nifurtimox-eflornithine combination therapy is no worse than that of eflornithine monotherapy [61]. However, this combination therapy represents a major advance in terms of making the treatment safer, cheaper and easier to administer [61].

## Conclusion

The development of the anti-sleeping sickness drugs is an early example of the employment of medicinal chemistry, the stepwise application of structure-activity relationships in order to increase the trypanocidal activity of a lead compound and to simultaneously reduce toxic side effects by modifying its chemical structure. Rational drug design, the inventive process of finding new drugs based on the knowledge of a biological target, has so far produced only one drug for treatment of sleeping sickness, eflornithine, although this drug was initially developed as an anti-cancer agent. Thus, it seems that the traditional methods of medical chemistry may be more effective in the development of new chemotherapies for this important disease.

## Competing interests

The author declares that he has no competing interests.

## References

[B1] Molyneux DH Pentreath V Doua F Cook GC African trypanosomiasis in man Manson's Tropical Diseases 1996 20 London: W.B. Saunders 1171 1196

[B2] World Health Organization Control of human African trypanosomiasis: a strategy for the African region 2005 AFRO, AFR/RC55/11

[B3] Kuzoe FA Current situation of African trypanosomiasis Acta Trop 1993 54 153 162 10.1016/0001-706X(93)90089-T 7902654

[B4] World Health Organization African trypanosomiasis (sleeping sickness) World Health Organ Fact Sheet 2006 259 http://www.who.int/mediacentre/factsheets/fs259/en/

[B5] Steverding D The history of African trypanosomiasis Parasit Vectors 2008 1 3 10.1186/1756-3305-1-3 18275594 PMC2270819

[B6] Bosch F Rosich L The contributions of Paul Ehrlich to Pharmacology: a tribute on the occasion of the centenary of his Nobel Prize Pharmacology 2008 82 171 179 10.1159/000149583 18679046 PMC2790789

[B7] Williamson J Mulligan HW Review of chemotherapeutic and chemoprophylactic agents The African trypanosomiasis 1970 London: Allen & Unwin 125 221

[B8] Travis AS The rainbow makers: the origins of the synthetic dyestuff industry in Western Europe 1993 Bethlehem, PA: Lehigh University Press

[B9] Sneader W Drug discovery: a history 2005 Chichester: John Wiley

[B10] Travis AS Paul Ehrlich: a hundred years of chemotherapy 1891-1991 Biochemist 1991 13 9 12

[B11] Ehrlich P Shiga K Farbentherapeutische Versuche bei Trypanosomenerkrankung Berl Klin Wochenschr 1904 41 329 332, 362-365

[B12] Ehrlich P Chemotherapeutische Trypanosomen-Studien Berl Klin Wochenschr 1907 44 233 236, 280-283, 310-314, 341-344

[B13] Nicolle M Mesnil F Traitement des trypanosomiases par les couleurs de benzidine. Premiére partie - etude chemique Ann Inst Pasteur 1906 20 417 448

[B14] Mesnil F Nicolle M Traitement des trypanosomiases par les couleurs de benzidine. Second partie - etude expérimentale Ann Inst Pasteur 1906 20 513 538

[B15] Dressel J Oesper RE The discovery of Germanin by Oskar Dressel and Richard Kothe J Chem Edu 1961 38 620 621 10.1021/ed038p620

[B16] Pope JW Synthetic therapeutic agents Br Med J 1924 1 413 414 10.1136/bmj.1.3297.413 PMC2303898 20771495

[B17] Fourneau E Tréfouël J Vallée J Recherches de chimiothérapie dans la série du 205 Bayer. Urées des acides aminobenzoylaminonaphtaléniques Ann Inst Pasteur 1924 38 81 114

[B18] Livingston D Arsenic as a remedy for tsetse bite Br Med J 1858 70 360 361 10.1136/bmj.s4-1.70.360-a

[B19] Lavaran A Mesnil F Trypanosomes et Trypanosomiases 1904 Paris: Masson et Cie

[B20] Thomas HW The experimental treatment of trypanosomiasis in animals Proc Roy Soc Ser B 1905 76 589 591 10.1098/rspb.1905.0051

[B21] Jacobs WA Heidelberger M Aromatic arsenic compounds v. *N*-substituted glycylarsanilic acids J Am Chem Soc 1919 41 1809 1821 10.1021/ja02232a012

[B22] Vickerman K Hide G, Mottram JC, Coombs GH, Holmes PH Landmarks in trypanosome research Trypanosomiasis and Leishmaniasis. Biology and Control 1997 Wallingford, Oxon: CAB International 1 37

[B23] Friedheim EA L'acide triazine-arsinique dans le traitement de la maladie du sommeil Ann Inst Pasteur 1940 65 108 118

[B24] Friedheim EA Some approaches to the development of chemotherapeutic compounds Ann Trop Med Parasitol 1959 53 1 9 13650482 10.1080/00034983.1959.11685892

[B25] Friedheim EA Melarsen oxide in the treatment of human trypanosomiasis Ann Trop Med Parasitol 1948 42 357 363 18110349 10.1080/00034983.1948.11685383

[B26] Friedheim EA Mel B in the treatment of human trypanosomiasis Am J Trop Med Hyg 1949 29 173 180 18116843 10.4269/ajtmh.1949.s1-29.173

[B27] Yorke W Adams ARD Murgatroyd F Studies in chemotherapy. I. A method for maintaining pathogenic trypanosomes alive *in vitro *at 37°C. for 24 hours Ann Trop Med Parasitol 1929 23 501 518

[B28] Poindexter HA Further observations on the relation of certain carbohydrates to *Trypanosoma equiperdum *metabolism J Parasitol 1935 21 292 301 10.2307/3271360

[B29] von Jancsó N von Jancsó H Chemotherapeutische Wirkung und Kohlehydratstoffwechsel: die Heilwirkung von Guanidinderivaten auf die Trypanosomeninfektion Z Immunitätsforsch Exper Ther 1935 86 1 30

[B30] Schern K Artagaveytia-Allende R Zur glykopriven Therapie und Prophylaxe mit sowohl toxisch als auch atoxisch wirkenden Substanzen bei der experimentellen Trypanosomen- und Treponemeninfektion Z Immunitätsforsch Exper Ther 1936 89 21 64

[B31] Lourie EM Yorke W Studies in chemotherapy. XVI. The trypanocidal action of synthalin Ann Trop Med Parasitol 1937 31 435 445

[B32] King H Lourie EM Yorke W Studies in chemotherapy. XIX. Further report on new trypanocidal substances Ann Trop Med Parasitol 1938 32 177 192

[B33] Lourie EM Yorke W Studies in chemotherapy. XXI. The trypanocidal action of certain aromatic diamidines Ann Trop Med Parasitol 1939 33 289 304

[B34] Ashley JN Barber HJ Ewins AJ Newbery G Self ADH A chemotherapeutic comparison of the trypanocidal action of some aromatic diamidines J Chem Soc (London) 1942 103 116

[B35] Lourie EM Treatment of sleeping sickness in Sierra Leone Ann Trop Med Parasitol 1942 36 113 131

[B36] Napier LE Sen Gupta PC A peculiar neurological sequel to administration of 4:4'-diamidino-diphenyl-ethylene (M&B 744) Indian Med Gaz 1942 77 71 74 PMC5169058 29012492

[B37] Collard PJ Hargreaves WH Neuropathy after stilbamidine treatment of Kala-Azar Lancet 1947 250 686 688 10.1016/S0140-6736(47)90716-2 20271209

[B38] Soeiro MNC De Souza EM Stephens CE Boykin DW Aromatic diamidines as antiparasitic agents Expert Opin Investig Drugs 2005 14 957 972 10.1517/13543784.14.8.957 16050790

[B39] Das BP Boykin DW Synthesis and antiprotozoal activity of 2,5-bis(4-guanylphenyl)furans J Med Chem 1977 20 531 536 10.1021/jm00214a014 321783

[B40] Steck EA Kinnamon KE Davidson DE Jr Duxbury RE Johnson AJ Masters RE *Trypanosoma rhodesiense*: evaluation of the antitrypanosomal action of 2,5-bis(4-guanylphenyl)furan dihydrochloride Exp Parasitol 1982 53 133 144 10.1016/0014-4894(82)90099-6 7056341

[B41] Boykin DW Kumar A Hall JE Bender BC Tidwell RR Anti-pneumocystis activity of bis-amidoximes and bis-O-alkylamidoximes prodrugs Bioorg Med Chem Lett 1996 6 3017 3020 10.1016/S0960-894X(96)00557-4

[B42] Wenzler T Boykin DW Ismail MA Hall JE Tidwell RR Brun R New treatment option for second-stage African sleeping sickness: *in vitro *and *in vivo *efficacy of aza analogs of DB289 Antimicrob Agents Chemother 2009 53 4185 4192 10.1128/AAC.00225-09 19620327 PMC2764217

[B43] Docampo R Moreno SN Stoppani AO Leon W Cruz FS Villalta F Muniz RF Mechanism of nifurtimox toxicity in different forms of *Trypanosoma cruzi* Biochem Pharmacol 1981 30 1947 1951 10.1016/0006-2952(81)90204-5 7023488

[B44] Pépin J Milord F Mpia B Meurice F Ethier L DeGroof D Bruneel H An open clinical trial of nifurtimox for arseno-resistant *Trypanosoma brucei gambiense *sleeping sickness in central Zaire Trans R Soc Trop Med Hyg 1989 83 514 517 10.1016/0035-9203(89)90270-8 2694491

[B45] Pépin J Milord F Meurice F Ethier L Loko L Mpia B High-dose nifurtimox for arseno-resistant *Trypanosoma brucei gambiense *sleeping sickness: an open trial in central Zaire Trans R Soc Trop Med Hyg 1992 86 254 256 10.1016/0035-9203(92)90298-Q 1412646

[B46] Metcalf BW Bey P Danzin C Jung MJ Casara P Vevert JP Catalytic irreversible inhibition of mammalian ornithine decarboxylase (E.C. 4.1.1.17) by substrate and product analogues J Am Chem Soc 1978 100 2551 2553 10.1021/ja00476a050

[B47] Meyskens FL Jr Gerner EW Development of difluoromethylornithine (DFMO) as a chemopreventive agent Clin Cancer Res 1999 5 945 951 10353725

[B48] Oredsson S Anehus S Heby O Inhibition of cell proliferation by DL-α-difluoromethylornithine, a catalytic irreversible inhibitor of ornithine decarboxylase Acta Chem Scan 1980 34B 457 458 10.3891/acta.chem.scand.34b-0457 6781187

[B49] Fairlamb AH Chemotherapy of human African trypanosomiasis: current and future prospects Trends Parasitol 2003 19 488 494 10.1016/j.pt.2003.09.002 14580959

[B50] Tabor CW Tabor H Polyamines Annu Rev Biochem 1984 53 749 790 10.1146/annurev.bi.53.070184.003533 6206782

[B51] Bacchi CJ Nathan HC Hutner SH McCann PP Sjoerdsma A Polyamine metabolism: a potential therapeutic target in trypanosomes Science 1980 210 332 334 10.1126/science.6775372 6775372

[B52] Van Nieuwenhove S Schechter PJ Declercq J Boné G Burke J Sjoerdsma A Treatment of gambiense sleeping sickness in the Sudan with oral DFMO (DF-α-difluoromethylornithine), an inhibitor of ornithine decarboxylase; first field trial Trans R Soc Trop Med Hyg 1985 79 692 698 10.1016/0035-9203(85)90195-6 3938090

[B53] Doua F Boa FY Schechter PJ Miézan TW Diai D Sason SR De Raadt P Haegele KD Sjoerdsma A Konian K Treatment of human late stage gambiense trypanosomiasis with α-difluoromethylornithine (eflornithine): efficacy and tolerance in 14 cases in Côte d'Ivoire Am J Trop Med Hyg 1987 37 525 533 3120607 10.4269/ajtmh.1987.37.525

[B54] Pépin J Milord F Guern C Schechter PJ Difluoromethylornithine for arseno-resistant *Trypanosoma brucei gambiense *sleeping sickness Lancet 1987 330 1431 1433 10.1016/S0140-6736(87)91131-7 2891995

[B55] Eozenou P Jannin J Ngampo S Carme B Tell GP Schechter PJ Essai de traitement de la trypanosomiase à *Trypanosoma brucei gambiense *par l'Eflornithine en République Populaire du Congo Med Trop (Mars) 1989 49 149 154 2507863

[B56] Iten M Mett H Evans A Enyaru JCK Brun R Kaminsky R Alterations in ornithine decarboxylase characteristics account for tolerance of *Trypanosoma brucei rodesiense *to D,L-α-difluoromethylornithine Antimicrob Agents Chemother 1997 41 1922 1925 9303385 10.1128/aac.41.9.1922 PMC164036

[B57] Nightingale SL Drug for sleeping sickness approved JAMA 1991 265 1229 1995961 10.1001/jama.265.10.1229

[B58] Priotto G Fogg C Balasegaram M Erphas O Louga A Checchi F Ghabri S Piola P Three drug combinations for late-stage *Trypanosoma brucei gambiense *sleeping sickness: a randomized clinical trial in Uganda PLoS Clin Trials 2006 1 e39 10.1371/journal.pctr.0010039 17160135 PMC1687208

[B59] Checchi F Piola P Ayikoru H Thomas F Legros D Priotto G Nifurtimox plus eflornithine for late-stage sleeping sickness in Uganda: a case series PLoS Negl Trop Dis 2007 1 e64 10.1371/journal.pntd.0000064 18060083 PMC2100371

[B60] Priotto G Kasparian S Ngouama D Ghorashian S Arnold U Ghabri S Karunakara U Nifurtimox-eflornithine combination therapy for second-stage *Trypanosoma brucei gambiense *sleeping sickness: a randomized clinical trial in Congo Clin Infect Dis 2007 45 1435 1442 10.1086/522982 17990225

[B61] Priotto G Kasparian S Mutombo W Ngouama D Gharashian S Arnold U Ghabri S Baudin E Buard V Kazadi-Kyanza S Ilunga M Mutangala W Pohlig G Schmid C Karunakara U Torreele E Kande V Nifurtomox-eflornithine combination therapy for second-stage African *Trypanosoma brucei gambiense *trypanosomiasis: a multicentre, randomised, phase III, non-inferiority trial Lancet 2009 374 56 64 10.1016/S0140-6736(09)61117-X 19559476

